# Fat Necrosis of the Breast After Coronary Artery Bypass Grafting Presenting Similarly to Inflammatory Breast Cancer

**DOI:** 10.7759/cureus.26524

**Published:** 2022-07-03

**Authors:** Caroline G Horne, Michael Breiner

**Affiliations:** 1 Department of Medicine, Edward Via College of Osteopathic Medicine, Blacksburg, USA; 2 Department of Surgery, Edward Via College of Osteopathic Medicine, Blacksburg, USA

**Keywords:** internal mammary artery, breast mass, malignancy, coronary artery bypass grafting, breast necrosis, breast cancer, inflammatory breast cancer, fat necrosis

## Abstract

The clinical presentation of a new breast mass warrants a broad initial differential diagnosis. With breast cancer as the most common female malignancy in the United States, it is critical to consider the various types of breast cancer in the differential diagnosis. However, there are also a myriad of benign etiologies for a new breast mass that also need to be considered. Fat necrosis of the breast is a benign condition that may be mistaken as malignancy and often develops as the result of trauma to the breast tissue. This report describes a case of fat necrosis of the breast in a patient that developed as a rare complication months after undergoing coronary artery bypass graft surgery that presented with symptoms concerning for possible malignancy.

## Introduction

Fat necrosis of the breast is a benign condition that can often cause some misperception diagnostically among clinicians. On physical examination, fat necrosis in the breast can often be confused with breast malignancy. This often prompts a more in-depth evaluation of patients, consisting of biopsies, mammograms, ultrasounds, and/or computed tomography (CT) in order to rule out cancer and support a definitive diagnosis of fat necrosis [1].

Fat necrosis is most often a result of accidental trauma; however, other etiologies include surgery, radiation therapy, or even unrecognized physical trauma. On physical exam, it presents as a painless, non-discolored subcutaneous lump with size usually correlating to the severity of the trauma. Approximately one-fourth of patients with fat necrosis will present with overlying bruising and tenderness. With respect to breast necrosis, only 14% of patients report skin dimpling and as few as 9% experience nipple inversion, both of which may also be signs of malignancy of the breast [2].

The underlying pathophysiology of fat necrosis in the context of accidental trauma consists of early bleeding into impacted adipose tissue which presents clinically as induration. The induration progresses over the course of days to weeks and results in clear demarcation of the necrotic region via yellow-red discoloration of the skin. With the continued passage of time, the hemorrhage within the adipose undergoes cystic degeneration to form an oil-filled cavity with or without the additional formation of calcifications. Eventually, the area of fat necrosis will undergo hemosiderin deposition and fibrosis, though the cystic element may continue for months or years [2].

The initial differential diagnosis of a breast mass can be quite extensive, consisting of both benign and malignant pathologies; however, the key action in these patients is to rule out breast malignancy [3]. Breast cancer is the most frequently diagnosed malignancy and the leading cause of cancer death in women across the globe. Specifically in the United States, breast cancer is the most common female malignancy and the second most common cause of cancer death in women [4]. Today, most patients diagnosed with breast cancer initially have the malignancy detected through abnormal screening mammography. Nonetheless, approximately 15% are diagnosed by the presence of a breast mass that was not visualized on a mammogram and an additional 30% of patients detect a new breast mass in the time between mammogram screenings. The mass that develops in the breast related to breast cancer is often noted to be immobile, firm, and singular with irregular borders [4].

Inflammatory breast cancer is one of the more uncommon types of breast cancer, making up only 0.5 to 2% of breast cancer diagnoses in the United States; however, it is critical to include on the differential diagnosis of a breast mass. Inflammatory breast cancer is a very aggressive form of breast cancer that is often diagnosed in Caucasian women around 59 years of age who present with a tender, rapidly enlarging breast mass with overlying skin changes noticed on self-examination. The skin overlying the mass has a peau d’orange appearance and is thickened, warm, and has a red-purple discoloration resembling ecchymosis [5]. In any patient presenting with breast mass suspicious for malignancy, a biopsy of the mass should be completed, often via image-guided core needle biopsy [3]. Importantly, if there is a concern for inflammatory breast cancer, then skin biopsies must also be obtained in addition to the core needle biopsy of the mass in order to either make or rule out the diagnosis [5]. Benign etiologies to consider in a patient presenting with a mass of the breast tissue include fibroadenoma, simple cyst, fibrocystic changes, an abscess, or fat necrosis [3].

## Case presentation

A 69-year-old Caucasian female with a past medical history of diabetes mellitus, coronary artery disease, and hypertension presented to the general surgery clinic with a complaint of a new mass in her left breast at the eight o’clock position. She first noticed the mass approximately two months prior and stated it had been gradually increasing in size. The patient endorsed performing regular self-breast exams and felt confident in her description of the timeline with respect to the emergence and growth of the mass. She reported tenderness in the region of the mass and overlying skin changes in the region consisting of erythema and bruising. She denied any nipple discharge, inversion of the nipple, change in weight, change in appetite, and fever or chills.

Her obstetric history included two full-term pregnancies with her first child being born when she was 30 years of age. She denied breastfeeding either child. Her gynecologic history consisted of onset of menarche at age 14 and onset of menopause at age 59. She had a history of intermittent hormonal birth control use for approximately 15 years. Her most recent pap smear was normal two years ago.

She denied a family history of breast, ovarian, or colon cancer, though did have a positive family history of ischemic heart disease. She had a surgical history consisting of coronary artery bypass grafting (CABG) of the left anterior descending artery, left circumflex artery, and proximal right coronary artery approximately two months prior. She also had a surgical history of bilateral breast reduction in 1975, tubal ligation, hysteroscopy, spinal surgery, and sigmoid surgery. Her social history was notable for a 50-pack-year tobacco use, but the patient denied significant use of alcohol or illicit drugs. All of her mammography screenings prior to the recognition of this new breast mass, including prior to her CABG surgery, had been unremarkable.

The patient’s vital signs were all within normal limits. On the general physical exam, there was a notable well-healed median sternotomy scar from her recent CABG procedure; however, the rest of the exam was unremarkable. Her breast exam revealed bilateral scars from her prior breast reduction surgery, a firm palpable mass approximately 6 cm x 4 cm in size located at the lower inner quadrant of the left breast with overlying mottled skin changes and skin dimpling. Additionally, a 2-cm axillary lymph node was palpable on the left side. The right breast exam was within normal limits.

**Figure 1:**
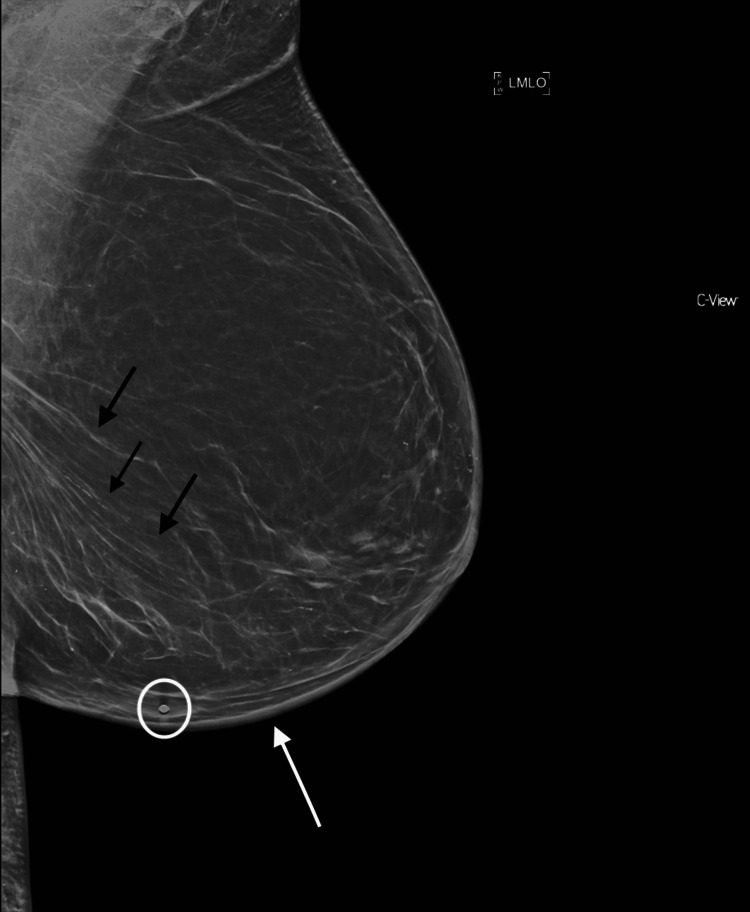
Mammography of Left Breast: Left Mediolateral Oblique View This mammogram of the left breast, taken prior to a core needle biopsy, reveals mild increased diffuse skin thickening (white arrow) along the dependent portion of the breast with additional trabecular thickening (black arrows) and small, radiopaque imaging marker (white circle).

**Figure 2:**
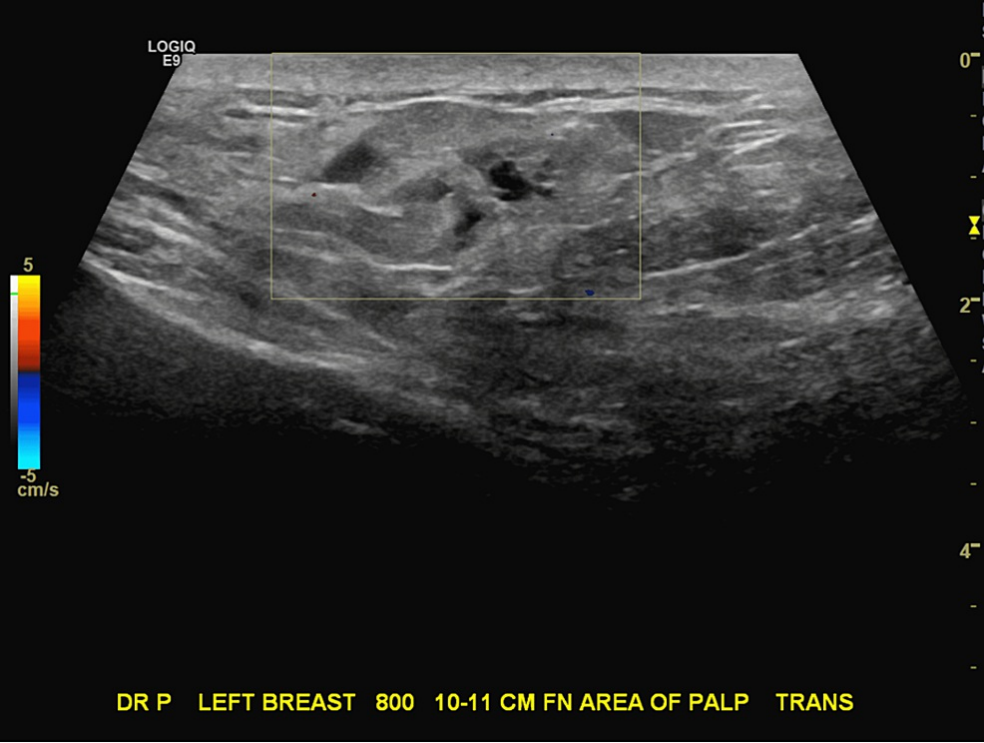
Ultrasound of Left Breast Mass with Color Doppler (Transverse View) This focused Doppler ultrasound image (transverse view) of the palpable left breast mass, taken approximately 10-11 cm from the nipple at the eight o'clock position, reveals a prominent fat lobule measuring 6 cm x 2 cm with an area of hypoechoic change along the edge of the lobule that is avascular and has the sonographic appearance of probable fat necrosis.

**Figure 3:**
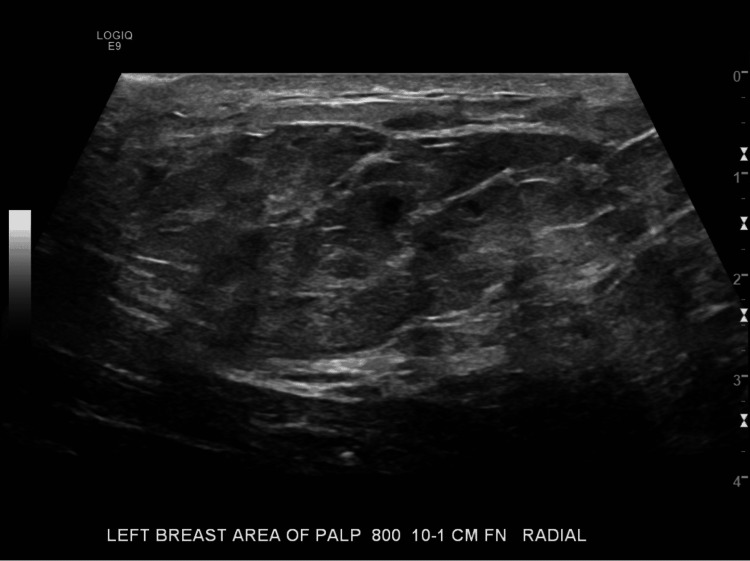
Ultrasound of Left Breast Mass (Radial View) This focused ultrasound image (radial view) of the palpable left breast mass, taken approximately 10-11 cm from the nipple at the eight o'clock position, reveals a prominent fat lobule measuring 6 cm x 2 cm with an area of hypoechoic change along the edge of the lobule that is avascular and has the sonographic appearance of probable fat necrosis.

One month prior to her presentation in the surgical clinic, the patient obtained a mammogram that revealed mild diffuse skin thickening, trabecular thickening bilaterally, skin thickening noted symmetrically along the dependent portions of both breasts, and a normal right breast (Figure 1). Additionally, she obtained a Doppler ultrasound of the palpable left breast mass that revealed a prominent fat lobule measuring 6 cm x 2 cm in the left breast along with an area of hypoechoic change that is avascular and has the sonographic appearance of probably fat necrosis (Figures 2, 3). The radiologic impression was Breast Imaging Reporting and Data System (BIRADS) category three suggesting probably benign findings with a recommendation for referral to surgery for correlation with a clinical breast exam.

Following her initial presentation to the surgery clinic, the patient underwent an ultrasound-guided core needle biopsy of the breast mass with an additional overlying punch skin biopsy, a follow-up mammogram, follow-up axillary lymph node exam, and a CT of the thorax.

The CT of the thorax revealed an asymmetric subcutaneous fatty protuberance between the left nipple and sternotomy that may be an area of concern and mild subcutaneous inflammatory changes suspected to be closely associated with the protuberance and chest wall which may be related to recent sternotomy. The post-biopsy mammogram of the left breast revealed appropriate post-procedural changes with radiologic recommendation for excisional biopsy of the left breast mass. The axillary lymph node ultrasound revealed a fatty replaced 4 cm x 3.1 cm reniform left axillary lymph node with a radiologic impression of BIRADS two, benign finding. The breast core needle biopsy results were classic for fat necrosis and the overlying skin biopsy results revealed congested vessels.

The final diagnosis for this patient based on tissue pathology and imaging was fat necrosis of the lower inner quadrant of the left breast status-post recent median sternotomy for three-vessel CABG sacrificing the left internal mammary artery and right saphenous veins with bilateral changes related to past bilateral reduction mammoplasty.

On follow-up one-week status-post left breast mass biopsy, the patient’s breast mass had increased in size to 11 cm x 10 cm. There was no definitive explanation for the continued increase in the size of the breast mass; however, the increase was suspected to be due to persistent inflammation from the CABG surgery and recent breast biopsies. No interventions were performed at this follow-up, and it was decided to proceed with observational management given the reassuring pathology results from her breast biopsy. The patient continued to follow up regularly for assessment of the breast mass which slowly began to decrease in size spontaneously, as well as follow-up for repeat mammography. Excisional biopsy was deferred at the time due to concern for increased risk of infection due to a recent median sternotomy.

## Discussion

Breast necrosis secondary to the use of the internal mammary artery for CABG revascularization is a rare complication of the procedure due to the rich vasculature of the breast. More commonly, complications status post CABG procedure include infection of sternotomy site with mediastinitis, osteomyelitis, and bleeding or cardiovascular/perfusion complications [6]. The internal mammary arteries are responsible for supplying the breast tissue and the anterior chest wall bilaterally; however, it also happens to be one of the most commonly utilized vessels for CABG procedures. The breast tissue, being highly vascularized, also receives additional blood supply from branches of the axillary artery and the intercostal arterial system. Fat necrosis of the breast that presents after surgical procedures occurs most often in the setting of a breast surgery; however, it can be associated with any surgery that alters the internal mammary artery [7].

Ischemia leading to fat necrosis of the breast is an uncommon malady that requires detailed work up to rule out potentially serious malignant etiologies for a new breast mass. Patient risk factors for breast malignancy also need to be considered in the workup and in creating the differential diagnosis for a patient with a new breast mass. There are various components that are associated with an increased risk for breast cancer development, including increased age, female sex, white race, and increased lifelong exposure to estrogen. Situations that would impact a woman’s cumulative exposure to estrogen include obesity and metabolic syndrome, onset of menarche, onset of menopause, history of oral hormonal replacement or contraception, gravity and parity, and age at first full-term pregnancy. There are also known genetic risk factors for breast cancer including a positive family history of BRCA1/2 mutations and a family history of familial cancer syndromes [8]. This patient presented with concerning symptoms of breast malignancy, including new-onset rapidly enlarging mass, overlying skin thickening, skin dimpling, overlying erythema, enlarged axillary lymph nodes, and uncertain tissue changes on mammography (BIRADS 3). Additionally, she had risk factors for breast malignancy, such as tobacco use, obesity, increased age at first full-term pregnancy, advanced age, Caucasian race, and female gender.

Breast cancer is the second leading cause of cancer-related deaths in American women and the most common cause of cancer for women in the USA [4]. The most common breast cancer is infiltrating ductal carcinoma which accounts for up to 70-80% of breast cancers, followed by infiltrating lobular carcinoma [4]. Inflammatory breast cancer is a rare, but aggressive form of breast cancer making up only 0.5-2.0% of breast cancer diagnoses; however, it is critical to include a differential diagnosis for breast pain or mass due to its particularly poor prognosis. Inflammatory breast cancer may present similarly to benign etiologies of breast pain or breast mass, such as mastitis or fat necrosis. It often appears as breast pain or a rapidly growing lump that is tender and firm with associated overlying skin warmth, redness, and thickening [5]. Unfortunately, at the time of patient presentation, the malignancy often has already extended to involve the lymphatic system in most patients, with distant metastasis present in about approximately 33% of patients [9]. Therefore, all healthcare providers should maintain high clinical suspicion for any new onset breast pain or breast mass, although the resulting etiology may be benign.

Fortunately, after a thorough cancer workup was negative, this patient was diagnosed with fat necrosis of the breast secondary to tissue ischemia resulting from use of the left internal mammary artery during a recent CABG procedure. The treatment for fat necrosis consists mainly of surveillance, as the process is usually self-limiting and resolves spontaneously over time [10]. Observational management is sufficient for fat necrosis as long as the patient does not complain of intolerable pain and cosmetic appearance is not of significant concern at that time [7]. On occasion, surgical intervention may be considered for biopsy-confirmed breast necrosis that has continued to increase in size, has associated pain, or fails to resolve spontaneously resulting in cosmetic concerns [7, 10]. However, if suspected fat necrosis of the breast in a patient who initially elected to proceed with observational management does not begin to improve within six months, then a full work up for breast mass should be completed to rule out malignancy, including mammogram and additional biopsy of the lesion as indicated [7].

## Conclusions

This case study highlights the similarities in patient presentation for breast malignancy and benign fat necrosis of the breast, particularly the similarities between the presenting symptoms of fat necrosis and inflammatory breast cancer. Additionally, this report brings attention to fat necrosis of the breast as a rare complication of coronary artery bypass graft surgery. This case supports the notion that providers should maintain high clinical suspicion for inflammatory breast cancer in cases of suspected fat necrosis of the breast due to the extremely poor prognosis of the malignancy in general, particularly if diagnosis is delayed.

Similar cases should be managed based on the provider's best clinical judgement. Fat necrosis is often a self-limiting condition that slowly resolves spontaneously over time; therefore, observational management is appropriate for biopsy-proven fat necrosis or for patients with a breast mass that is highly suspicious for fat necrosis after obtaining a thorough history and physical exam. Surgical excision may be completed based on patient preferences for fat necrosis of the breast that fails to resolve. It is suggested that providers maintain a low threshold for proceeding with mammography with or without breast mass biopsy in patients with suspected fat necrosis or in patients presenting with breast mass with concerning features and/or significant risk factors for breast malignancy.
